# Vitamin C therapy in septic shock

**DOI:** 10.1186/s13054-022-03965-7

**Published:** 2022-03-30

**Authors:** Syed Nabeel Muzaffar, Sai Saran, Suhail Sarwar Siddiqui

**Affiliations:** Department of Critical Care Medicine, King George’s Medical University, Lucknow, Uttar Pradesh 226003 India

To the Editor

We read with great interest the article by Patrick Rosengrave et al. [1], published recently in your esteemed journal, on intravenous (IV) vitamin C therapy in sepsis. According to this study, there was no significant improvement in vasopressor response with vitamin C. However, we would like to draw attention to a few points:

First, Zabet and colleagues [2], who found a beneficial favorable vasopressor response with vitamin C, included mainly less sick and postoperative critically ill patients. As vitamin C therapy surprisingly failed to show any significant improvement in vasopressor response in the sicker population studied by Rosengrave et al. [1], the overall effect of vitamin C at this dosage on vasopressor response may be questioned. Earlier assays used to determine vitamin C were based on colorimetric assays, which tend to overestimate vitamin C levels. So, it is possible that cut offs for hypovitaminosis C may be even lower than what is usually considered as deficient, although this possibility remains largely speculative. Moreover, around 85% of study population was from Europe, and baseline levels of vitamin C may vary across regions or have an inter-individual variation.

Second, neuronal cells and activated leukocytes actively concentrate vitamin C via sodium-dependent SVCT2 transporter, which protects them from reactive oxygen species. Besides serum concentrations, it may be essential to monitor the tissue concentrations of vitamin C (e.g., adrenal vitamin C levels for vasopressor synthesis) to ascertain the adequacy of vitamin C therapy. However, how tissue vitamin C levels would influence serum vitamin C levels and thus translate to clinical benefit needs further research.

Third, limited research has been conducted on pharmacokinetics of vitamin C. Plasma levels vary with route of administration, dose and duration of therapy, half-life of vitamin C, and underlying renal clearance (glomerular hyperfiltration or renal failure).

Fourth, hypovitaminosis C may also be an adaptive response to severity of illness, as evident from higher C-reactive protein (CRP) levels and neutrophil counts in vitamin C group in this study, which may reflect the underlying inflammatory stress. However, it is also possible that the link between vitamin C and CRP levels may just be a random effect.

Fifth, vitamin C may have a beneficial effect on microcirculation and tissue perfusion in sepsis, as shown in a recent study by Lavillegrand et al. [3]. Although no beneficial effect on vasopressor response was noticed in this study, it would have been interesting to look for an improvement in microcirculation.

Sixth, most of the patients had an underlying abdominal etiology (35%) of sepsis, requiring adequate source control measures, appropriate antibiotics and adherence to other sepsis bundle components for an improvement in hemodynamics. Moreover, time to ICU admission, duration of illness, inflammatory versus anti-inflammatory phase of sepsis also have an important bearing on effects of vitamin C.

Finally, thiamine is an essential co-factor in various important metabolic pathways in humans and low thiamine levels may be associated with poor outcomes [4]. It would have been useful to check serum thiamine levels in these patients as thiamine deficiency often shares the same risk factors as those of vitamin C deficiency. However, supplementation of thiamine may need individualization as some studies have also highlighted as well a rather negative role of thiamine supplementation [5].

Many studies are turning up in the area of vitamin C therapy in sepsis, but most of them have failed to meet the pre-defined end points. Hence, further research is required on the role of vitamin C therapy in hemodynamic optimization in sepsis.

## Data Availability

Not applicable.
